# “If the funders said to me that I have to dress in orange, I would dress in orange”: findings from qualitative interviews on researchers’ experiences of public involvement

**DOI:** 10.1186/s40900-026-00901-9

**Published:** 2026-07-03

**Authors:** Lili Gyopár, Kirsten Bell, Viveka Guzman, Habiba Haque, Amna Rida, Paapa Baffoe, Dave Varughese, Helen Ward

**Affiliations:** 1 Patient Experience Research Centre (PERC), School of Public Health, Imperial College, London, UK; 2 PERC community partner, London, UK; 3 Imperial College School of Medicine, London, UK

**Keywords:** Patient and public involvement, Qualitative interviews, Researchers’ views, Impact

## Abstract

**Background:**

Public involvement is increasingly mandated in health and medical research in the UK. With a push for its implementation from major funding bodies over the past few decades, we aimed to understand its current role in research through the lens of researchers – has it become embedded into their research or is it mostly a tokenistic exercise? In this qualitative interview project, we add to the growing body of literature on this topic by exploring researchers’ experiences with public involvement to better understand their views, motivations and attitudes towards these practices.

**Methods:**

In 2025, 15 semi-structured online interviews were conducted with biomedical researchers in the NIHR Imperial Biomedical Research Centre about their experiences of public involvement. The interviews were conducted for evaluation purposes and were carried out by undergraduate medical students as part of their research methods training. Thematic and inductive analysis was used to identify key themes across the data. The project was supported by a community partner, who helped in the development of the interview guide and data analysis.

**Results:**

Funding mandates played a significant role in researchers’ motivations for conducting public involvement and appear to have created a distinction between an imposed model of public involvement, in which it is ‘unreflective’ and ‘box-ticking’, and an organic model in which it is ‘sincere’ and ‘meaningful’. Participants highlighted the discrepancies between funders’ expectations and the realities of academic research, including the limited resources available for public involvement. The feasibility and desirability of public involvement in different research areas was also questioned, especially in non-clinical research. Although researchers frequently reported that doing public involvement made them see the value of it, most of the impacts they identified were intangible rather than tangible.

**Conclusions:**

Strengthening meaningful public involvement requires further investment from funders and institutions, along with a more tailored approach for different research areas rather than a ‘one-size-fits-all’ orientation. We support calls for an expanded view of impact that treats it as a dialogue between researchers and the public rather than an intervention with measurable effects.

**Supplementary Information:**

The online version contains supplementary material available at 10.1186/s40900-026-00901-9.

## Abstract (plain language summary)

Public involvement is increasingly required to receiving funding for health and medical research in the UK. However, it is unclear whether researchers see public involvement as desirable or whether they do it because they are told to. In this study, we carried out 15 interviews to understand UK researchers’ experiences with public involvement and their views, motivations and attitudes towards the topic. Our project highlights the difference between meaningful and tick-box public involvement. Researchers pointed to the mismatch between funders’ expectations and the realities of research, and the ways this undermined meaningful involvement. They also questioned the value of public involvement for all types of research. Although many researchers reported that doing public involvement made them see its significance, the impacts they identified were not concrete. We encourage funders and academic institutions to increase the support they provide for public involvement, consider a more tailored approach rather than a ‘one-size-fits-all’ standard for public involvement, and recognise a wider range of impacts.

**Supplementary Information:**

The online version contains supplementary material available at 10.1186/s40900-026-00901-9.

## Background

Today, public involvement in health research is viewed as essential to health and social care research. Although it goes by various names, including “patient and public involvement” (PPI), “patient involvement”, “patient and public involvement and engagement” (PPIE), and “patient and public involvement, engagement and participation” (PPIEP), it involves research being carried out ‘with’ or ‘by’ members of the public rather than ‘to’, ‘about’ or ‘for’ them, and encompasses patients, carers, service users and representatives [1, 2].

The National Institute for Health and Care Research (NIHR), which is funded by England’s Department of Health and Social Care, was an early leader in the field of public involvement, both in the UK and internationally. Based on the premise that incorporating public perspectives will democratise research and care, and improve its quality and impact, it has mandated public involvement for almost two decades [1]. In 2022, the UK Research & Innovation councils followed suit in declaring their commitment to improving public involvement in research. Their joint statement, titled “Putting People First”, asserts that “Public involvement is important, expected and possible in all types of health and social care research” [3].

In addition to its widespread implementation in clinical research, there is a growing push to incorporate public involvement in non-clinical research as well [4]. For example, in 2024, the Medical Research Council and NIHR developed a funding opportunity titled “Understanding public involvement in non-clinical research”, which sought to “improve the understanding, design and practice of public involvement in non-clinical health and biomedical research” [5].

Despite funders’ mandates to involve the public in the design of health and medical research, evidence of the impacts of public involvement on research outcomes remains sparse, although calls for such have repeatedly been made over the past fifteen years [1, 6–8]. Although positive effects on recruitment and research design have been found, studies suggest that the impacts of public involvement are complex rather than straightforward [6, 7, 9].

Prior qualitative studies point to a degree of ambivalence on the part of researchers about public involvement. This research highlights the potential clash between the needs, values and aspirations of researchers and the public, and the difficulties of reconciling academic expertise with “expertise by experience” [7, 10–14]. Concerns have also consistently been raised about tokenistic approaches to public involvement [7, 15, 16] and its tendency to become what Komporozos-Athanasiou and colleagues [13] term a “political ritual”.

Our aim in this article is to contribute to the growing number of empirical studies on researchers’ experiences of public involvement to better understand how these practices have been taken up and to what effect. In particular, has public involvement become standard practice in health and medical research, and has the NIHR’s goal [1] of moving beyond public involvement as a “tokenistic exercise” (p. 21) been achieved?

## Methods

This project used semi-structured interviews to explore the experiences of public involvement among researchers linked with the NIHR Imperial Biomedical Research Centre (BRC): an NIHR-funded research centre hosted by Imperial College Healthcare NHS Trust in partnership with Imperial College London – a global leader in health and medical research. As Boaz and colleagues [10] note, BRCs “are designed to conduct ‘bench to bedside’ translational research, speeding up the process of bringing new treatments for patients from the laboratory to the clinic and they include a range of types of research and researcher” (p. 594).

Researchers within the BRC are supported by the public involvement team within the Patient Experience Research Centre (PERC), which is a core facility of the BRC and implements the BRC’s PPIEP strategy, comprising support and advice on public involvement in health research, and collecting impact on public involvement. The project was conducted by researchers in PERC (KB, VG) with the support of four second-year medical students (LG, AR, PB, DV), who completed the initial interviews as part of their Clinical Research and Innovation (CRI) module, which aims to give students applied research training. The second set of interviews were conducted by LG, who stayed on in PERC as a project intern.

As we aimed to support undergraduate medical students’ learning about public involvement as well as qualitative methods, a PERC community partner (HH) supported the project throughout. Based on the GRIPP2-SF reporting checklist [17], her role was to ensure that students learned about public involvement from the perspective of public partners as well as researchers themselves. Her input informed the development of the project interview guide, and she was involved in the analysis throughout, including: (1) the preliminary stage when students were analysing the initial results, (2) the completion of the interviews when the analysis was being refined, and (3) the drafting of the paper. Her input throughout the project provided a critical counterpoint to the perspectives the researchers presented, and helped the students to understand what good public involvement looks like.

Recruitment for the project was supported by PERC’s public involvement team using their records of researchers who had previously requested support for public involvement activities. They made the initial approach to researchers in an email invitation explaining that we were interested in learning more about “your experiences of incorporating public involvement into your research.” The invitation further noted that the project was being conducted for quality assurance purposes and to support the skill development of the students. Those interested in taking part in an interview were asked to follow up directly with KB.

Twenty researchers were invited to participate, based on the public involvement team’s knowledge of which researchers had been funded to complete research projects, and 15 semi-structured interviews were carried out between June and August 2025 (*see* Table 1*for participant characteristics*). Following training in qualitative methods by KB and VG, and key informant interviews with two members of the public involvement team to gain critical background information about public involvement at Imperial, the student interns (LG, AR, PB, DV) carried out the interviews, with the majority carried out by LG (*N* = 10).

To accommodate researchers’ schedules, all interviews took place virtually on Microsoft Teams and lasted 25 min on average (range: 16–40 min). The interview guide was developed by the students, with support from KB, VG and HH, and was designed to take no longer than half an hour to accommodate the busy schedules of the interviewees. Topics covered participants’ understanding of public involvement and their experiences of the practice, including how they have incorporated it into their work and how it has impacted their research (see Appendix 1). In addition to the email consent for the interviews, verbal consent was obtained for the recording, and transcripts of the interviews were generated through Teams. The transcripts were then manually corrected and deidentified, and the recordings were deleted.

The interview transcripts were analysed thematically and inductively, based on a constructivist orientation that acknowledged the active role of the interviewer in producing their data [18], and influenced by analytic techniques drawn from critical discourse analysis [19]. To elaborate, central to our analysis was an awareness that public involvement is a *discourse* as well as a *practice*, and that researchers’ accounts were narratives that needed to be situated in the broader social structures within which public involvement is enacted. Published literature on researchers’ experience of public involvement served to orient the analysis and all members of the project team (LG, KB, VG, AR, PB, DV) read through the original interviews to look for key patterns and develop the coding frame, with the themes developed in dialogue with HH. Following the first set of interviews, the original codes and themes were revised by KB, LG and VG following the completion of further interviews and further discussion with HH.

At the outset of the project, we sought advice from the Imperial College London Research Governance and Integrity Team (RGIT) on whether institutional ethics review was required. Although the project was being carried out as part of PERC’s mandate to evaluate the impact of public involvement activities, we were concerned that the involvement of the students as part of their methods training would move this into the category of research requiring review. We were informed in writing that the project fell under the category of quality improvement and assurance and that institutional ethics approval was not required. However, good ethical practice was followed throughout, based on the professional ethical standards outlined by the UK Association of Social Anthropologists [20]. Considerable care was taken to ensure that participants did not feel coerced to participate based on their existing relationship with the PERC public involvement team. This was facilitated by the project being undertaken by PERC research staff and student interns, ensuring that the public involvement team were not privy to who ultimately participated.

Given the context of the interviews, there was no intent at the outset to publish the project findings. It was only upon completion of the original six interviews that we realised that interviewees’ reflections and insights might be of broader interest to practitioners in the field of public involvement, and that our project contributed to these larger conversations. Although publication is not the litmus test for whether institutional ethics review is required [21], and the intent of the project had not changed, we nevertheless recontacted the original interviewees to obtain their explicit permission to include their data in a publication; all consented to this usage. The possibility of publication was also made explicit in interview invitation for the subsequent nine interviews to ensure that their consent was fully informed. Following the completion of the manuscript, a draft was circulated to all interviewees for feedback and comment. Although we took considerable care to ensure that there were no details that might indirectly identify interviewees, we specifically asked them to confirm this when they reviewed the draft. One interviewee did request some minor wording changes at this point. Overall, the feedback we received was very positive and reaffirmed our sense that this remains an important topic to address. All names used in the article are pseudonyms.

**Table 1:** Participant characteristics

Characteristics	
**Gender**	
Women	9
Men	6
**Ethnicity**	
White	11
Other ethnicities	4
**Role**	
Mid-career or senior academic	4
Clinical research fellow	6
Research/research support staff	5

## Results

### The significance of public involvement mandates

Funders’ mandates for public involvement formed an unavoidable part of the conversation with researchers, with the topic broached either directly or indirectly in all but one interview. Most commonly, funders were mentioned when participants were asked about their motivations for public involvement. The majority of interviewees cited funding requirements; others simply went straight to talking about their grant applications. For example, one participant mentioned her fellowship six times throughout her interview.

Although the role of funders was widely discussed in explaining the impetus for public involvement, participants differed in the degree to which they highlighted its significance. Some acknowledged the role of funding but emphasised that this was not their primary motivation for doing public involvement. For example, Rapha, a clinical research fellow, noted, *“we’ve tried to include lived experience in our study*,* partly because it’s a requirement of funders*,* but mainly to make sure the research that we’re doing is relevant and is answering the questions that people want to hear.”* Likewise, Isabella, a clinical research fellow, stated, *“it wasn’t the funding that made us do them [public involvement activities]. It was more that we were like*,* ‘we want to do this’”*.

A minority indicated that their motivations were driven primarily by funding dictates. John, a senior academic, was the most explicit about this. In his words,

Umm, so we just—I mean, look, to be very frank about it, when you are in this game, the only thing that ultimately—the only thing you need to do to survive is to raise money. And if I’m completely frank about it, the reason I—we—involve patients in research is because we know that the funders wouldn’t fund any project without it. So honestly, if the funders said to me that every day I have to dress in orange, I would dress in orange. So, and I don’t mean to sound cynical, but that’s the world that we live in, like, everything revolves around getting money and if a funder says, ‘This is what you need to do to get money’, we don’t even think about whether this is right or wrong or good or bad, we just do it.

However, other researchers appeared more hesitant to express such views without encouragement, which came across in the following exchange in an interview with Stephanie, another senior academic:

Interviewer: And how important do you think public involvement is in your research?

Stephanie: [pauses, raises eyebrows, smiles and then laughs.] Wait, so—

Interviewer: “You can be completely honest.

Stephanie: [laughs] Yeah, I’ll tell you. You can’t get the grant or funding without them.

Embedded in these discussions of funding mandates was a distinction that numerous participants (*N* = 7) drew between ‘meaningful’, ‘sincere’ and ‘useful’ public involvement versus ‘unreflective’, ‘box-ticking’, ‘formulaic’ and ‘ratified’ versions done purely to satisfy funders’ demands. For example, when asked what the term public involvement meant to him, Roger, a clinical research fellow, responded, *“So*,* well*,* there’s two mean[ings]—there’s two words. One is it in its sincere and useful way*,* and the other one is in the way that it has to be done*,* in a way—in a sort of—what’s the right words here?—done in a sort of formulaic way to meet the expectations of funders.”* He indicated that he had done both “*meaningful PPI work*” and “*PPI for the sake of satisfying funders*,* even when it doesn’t seem sensible to me*”. Conversely, others spoke of box-ticking forms of public involvement as something that *other people* did. For example, Catherine, a clinical research manager, stated, *“I think for a lot of people*,* as I said*,* they just*,* they’re like ‘it’s a box to tick but I only think about it right at the end*,* just before I’m ready to submit everything’ and*,* ‘aargh*,* I haven’t done any PPI*,* what can I do? Oh*,* let’s just send the PIS [patient information sheet] out.’”*.

Notably, various participants (*N* = 5) highlighted that “informal” public involvement – i.e., interactions that researchers had with patients, members of the public and close friends which influenced their research in one way or another – was more valuable than formal public involvement, which served primarily to ratify the process. To quote Emilia, a clinical researcher:

I think the informal interactions are very important because I think that’s where, sometimes that’s where things will come naturally from patients and it’s not—they don’t answer a question that you ask and there’s no sort of bias introduced by the way you ask the question or, you know, by kind of what you’re trying to get out of the conversation. I think that’s—it’s a lot more organic and I think that feedback is really important… So I think that informal feedback is actually as important as the formal PPIE activities.

These responses speak to the widespread distinction interviewees invoked between an imposed model of public involvement, where it was expected regardless of whether it was feasible or desirable, and an organic model in which it was useful, valuable and improved their research.

### The tensions between public involvement and academic research

In response to questions about barriers to public involvement, ten researchers discussed at length how costly these activities were in labour, time and money, and the ways in which resource limitations and the structures of academic funding cycles often impeded their capacity to involve the public in their research in a meaningful way. For example, Sophia, a senior clinical researcher, stated that *“time and budget and capacity”* were critical to when and how researchers could involve the public in research. She noted that all three needed to be in place for public involvement to inform the inception of a research project, which was rarely possible. Jack, a clinical research fellow, similarly highlighted the significance of “time, costs, resource” and the limitations this posed on public involvement, *“given the tight time frames for research”*. Setting up public involvement, he noted, involved both *“cost and complexity”.*

The majority of our interviewees (*N* = 11) recognised the ways in which these resource limitations impacted which public contributors advised on research. Various participants discussed the tendency for the same voices to become overrepresented over time, significantly limiting the diversity and representativeness of those providing input. For example, Isabella (a research fellow) acknowledged that “you probably do end up with a less representative sample because you go back to the people that answer”.

Notably, funders’ requirements themselves were sometimes mentioned as active impediments to good public involvement, primarily because of the different temporalities of research funding cycles and public involvement activities. John reflected at length on this. In his words,

In an ideal world, we have lots of access to patients where we can speak to, and lots of time to do it. But the reality is that when a grant application, when a grant opportunity opens, you very often have like 6, 8 weeks to write the application… everything takes a huge amount of time and getting patient involvement needs to be done relatively quickly… the clock is so tight, that, you know, it’s really hard, basically.

Other researchers pointed to the paradox of public involvement *requiring* funding in order to *get* funding, which Emilia, a clinical research manager, described as a “*bit of a chicken and egg situation*”. To quote Arjun, a senior academic, “*I just wish there will be more support from universities and other funding bodies to*,* erm*,* sometimes it’s*,* you know*,* like*,* you can’t get the grant application without it*,* there’s no money to do it [laughs] upfront*,* you know. So it’s like a catch 22 situation [laughs]*”.

Nadia, a clinical researcher, also pointed out that the labour around public involvement was not rewarded academically. In her words,

I think the only thing I sort of reflected on was, oh, maybe I should be a bit careful about my time and reducing how much I do [laughs]. Because it’s, you know, I like doing it, but I did sort of think, ‘mental note to self: I’m being exclusively judged on my scientific output.’

While the majority of interviewees focused on the general tension between public involvement and the temporalities and structures of academic life, there was also a broad consensus that public involvement potentially makes less sense for non-clinical studies. For example, Sophia, who had started her career in non-clinical research and later moved into clinical research, summarised her experiences with both as follows: *“When I first started*,* as in research*,* I was—I suppose I was further away from the clinical end of it*,* so I didn’t really see the point of it as much as I do now. But obviously at the time I wasn’t—I was working with healthy controls and it wasn’t as relevant.”*

Bench scientists frequently questioned the relevance of public involvement to their research due to the lack of scientific knowledge of patients and the public and the complexity of their studies. In this context, they suggested, public involvement could not be anything except superficial. For example, Daisy, a mid-career academic, who described her research as “basic science”, explained the limited scope to involve the public in her research as follows:

I don’t, in all honesty, I don’t think it [public involvement] has changed my projects, because they’re really, really basic science… So, it, I don’t think it has changed [my research]. It has changed the wording of my projects and it has changed, you know, how I feel about them, I guess. But the projects themselves, I don’t think they have changed. Umm, no, in all honesty, they haven’t, but I think it’s because it really is basic science and it’s quite focused already.

For Daisy, it made sense to *engage* the public about her research, and she spoke very enthusiastically about this, but it did not make sense to *involve* them in its design. Arjun also pointed to the burdens this placed on members of the public, noting *“some areas may not need PPI or the group. It would be unfair for public to ask a specific question [small laugh]*,* very detailed*,* you know”.*

The overall view seemed to be that a more nuanced and realistic approach to public involvement by funders was required, which Stephanie described in terms of the pendulum having swung too far. “*I think we just need to wait for the pendulum to go in the middle again*”, she observed.

### The impact of public involvement

When asked how public involvement had influenced their research, participants described diverse effects that operated at different levels and timescales. This included concrete changes within their studies, as well as more intangible influences that shaped the ways they thought about or approached their research.

Among the tangible impacts described, several participants stated that public involvement had influenced the documents and strategies used for recruiting study participants, conducting their studies, or disseminating results. For instance, Rapha described multiple changes his team had made to the delivery of a study after feedback from their “*lived experience partners*”, such as increasing the number of therapy sessions, developing a booklet for study participants, and modifying their sample inclusion criteria. However, other participants highlighted that despite the positive effects of working together with public partners, these were not uniform across the research cycle. According to Nadia:

It would be disingenuous to pretend that, like, because of something a patient said, I’ve changed the aim of, like, my research questions—I haven’t really, because that’s really building on the science that I’ve generated. But it’s definitely kind of made me think about it in a different way.

Many participants (*N* = 10) mentioned that interactions with the public had shaped the ways they thought about their research but struggled to articulate the mechanisms and effects of this influence in a concrete way. For example, Sophia discussed at length the genuine openness of her team to public partners’ expertise and feedback, and the ways this had contributed to their research being “*more accessible*”, “*pragmatic*”, and “*sensitive to people’s needs*”, but observed that while it had “*absolutely changed what we do*,* I mean*,* it’s difficult to come up with specifics*”.

Meanwhile, other participants highlighted that public involvement had reaffirmed the value of their research and motivated them to continue. For instance, Daisy reported: *“you feel like your job and your day-to-day gains a bit of relevance—you feel useful”.* Grace echoed these perceptions and described it had helped her “*crystallise why I’m doing what I’m doing*”, to which she added: *“I guess I just really do think it improves the research that I’ve done. Erm*,* it’s humbling to hear how people have been affected by their illness or their child’s illness*,* and it’s very motivational to make me to carry on doing the work that I’m doing.”*

Some participants reported that collaborating with public partners was also akin to having cheerleaders for their research. Additionally, a few participants mentioned that conducting public involvement had enhanced their research skills and professional connections, as Grace illustrated: *“I think it’s a really meaningful skill to have developed and I can apply it in my future career in lots of different ways […] And*,* you know*,* some of the people*,* I will have long-term, professional*,* PPIE relationships with going forward*,* which is really nice.”*

Additionally, various interviewees mentioned that doing public involvement had made them see its actual value. For example, Felicia, a clinical research fellow, noted that doing public involvement “*really made me realise how important it is*”. Emilia likewise observed that *“I think it’s difficult until you see it*,* you experience it and you see the difference that it can make. I think it’s kind of difficult to convince a sceptic”.* These comments also point to the significance of “learning by doing” in public involvement. According to Bob, who had limited interactions with patients until his project:

Before we were doing this project … I didn’t have much idea [about public involvement]. So I have had a few conversations with patients, but it was all very informal and I didn’t know to what extent they could contribute. But now we form a better idea, so it’s — so we understand that patients can guide us into how we conduct the research or how we design our research, but also what sort of outcomes are — we should, not outcomes, but what sort of results we should aim to provide to patients and also like the — how that is very important to get their views in the kind of product that we are developing.

Finally, a few participants voiced concerns that funders’ framings of the impact of public involvement were not fit to capture many of the intangible effects outlined previously. For example, Roger described significant frustration with having to fit his conversations with public partners into *“something measurable and deliverable”* instead of focusing on true value, to which he added:

I need to try and steer this conversation really into something where it’s like looks very tangible impact. Even though it is having some impact, it’s just not measurable. I feel like I need to force in something measurable to tick the box for these funders, who want it to be demonstrated in a certain way, and I think that’s a shame.

## Discussion

Although participants had a range of experiences, opinions and attitudes regarding public involvement, funders’ requirements loomed large in the interviews and formed the inevitable backdrop to our conversations. Indeed, while public involvement is increasingly embedded in health and medical research, our interviews indicate that this is primarily because of funders’ mandates – a phenomenon noted in prior UK studies [12, 14]. As Boylan and colleagues [12] observe,

Pressure from funding bodies, and especially a strong steer from the NIHR, has undoubtedly fostered a climate in which PPI is seen as a requirement rather than an option. However, it is questionable whether this should be understood as truly ‘game-changing,’ or rather a stimulus to ‘gaming,’ in which ticking boxes are literally as well as metaphorically required” (p. 728).

Our findings suggest that funders’ expectations are a double-edged sword. By requiring public involvement in contexts where it might not have previously been considered, researchers are exposed to the practice and potentially come to appreciate its value. Certainly, a number of researchers we interviewed highlighted that *doing* public involvement made them see the *value* of it. However, it has clearly also led to the implementation of public involvement as more of a technocratic process than one that actively reshapes research relations [14, 16, 22–24]. As Cowden and Singh [25] warned almost two decades ago of the rise of user involvement in health and social care, “we are seeing the construction of a new hegemony in which progressive critiques have been incorporated into a system driven by managerial rather than democratizing imperatives” (p. 20).

A common thread in the interviews was that funding mandates had not been accompanied by adequate resourcing for public involvement – either by institutions or funders themselves. Notably, these challenges have been highlighted for decades [7, 10, 11, 15, 26–28]. For example, in 2009, Thompson and colleagues [26] observed that “The challenges of involving the public are well-documented. There are difficulties in obtaining adequate funding to involve the public meaningfully at the initial stages of research. The costs, in both time and money, appear to be key concerns” (p. 2010). Therefore, what is perhaps most striking about our findings is how *little* researchers’ concerns have changed over the past sixteen years, despite the fact that public involvement has become a formal fixture in UK health and medical research in the same period. However, this formalisation seems to have created a perception that only ‘ratified’ public involvement is valued by funders, although its organic, informal forms are often more useful.

Likewise, our findings regarding researchers’ reservations about the universal applicability and value of public involvement are far from novel [see 7, 12, 14, 29–31]. Indeed, prior studies have reported evidence of similar concerns about the necessity of public involvement in research at the ‘bench’ rather than ‘bedside’ end of the spectrum [e.g., 10, 26].

Although these concerns are typically treated as evidence of researchers’ unwillingness to share power based on perceived threats to their professional identity, a focus on individual researchers detracts from the broader structural constraints within which public involvement operates [29, 30]. Based on what participants told us, the widely discussed “cycle of tokenism” [12, 15] is not merely due to cynical researchers implementing public involvement in a half-hearted way that limits its effectiveness, thereby reinforcing their assumption of its lack of utility. Instead, it is partly due to the technical implementation of public involvement in the absence of a fundamental shift in academic culture and scientific norms, and a blanket approach that assumes that such activities are always appropriate, feasible or necessary.

Our findings therefore suggest that well-intentioned efforts to address problems of tokenism by distinguishing ‘involvement’ from ‘engagement’ may be serving to compound rather than address the problem. Rather than assuming that the public wants to be involved at the outset in all types of health and medical research, what may be required is a more nuanced approach that focuses on when public input can be meaningfully incorporated. Although conceptions of ‘meaningful’ public involvement may differ for public contributors and researchers themselves, tokenistic public involvement serves no one’s interests, especially the public partners who give their time and input with the expectation that it *will* make a difference. We therefore advocate the importance of research with public partners themselves about what they most want to accomplish from their involvement in research, and what type of research they want to be involved with.

This entails the need for a deeper consideration of the purpose of public involvement in research and whether its goals are democratic, technical or emancipatory – ideals that are potentially at odds and yet frequently treated as synonymous [12, 23, 27]. In particular, the question of whether public involvement is a means to an end or an end in its own right remains unresolved, although it has significant implications for how its impact is conceptualised [33].

This confusion about the purpose of public involvement was reflected in our interviews. In the rare instances where participants spoke in concrete terms about the impact of their public involvement, this typically related to improvements in the acceptability and feasibility of their existing study. However, researchers often struggled to identify what exactly had changed about their research, pointing in more general terms to the way it had changed their thinking and given them confidence in the value of their research – something also reported in prior studies [e.g., 12].

According to Russell and colleagues [33], driven by evidence-based medicine, approaches to public involvement that treat it as a means to an end typically position it as an intervention with a measurable effect size. This view, they argue, is appealing to researchers because it focuses on what is familiar to them (i.e., the scientific method) and the issue that primarily concerns them: namely, doing better research. Indeed, we saw evidence of this mentality amongst our interviewees, although they simultaneously doubted that the effects of public involvement were measurable. However, as Russell and colleagues [33] observe, this orientation tends to obscure other equally important conceptualisations of public involvement.

Staley and Barron [34] argue that the attempt to standardise involvement processes as ‘methods’ and to objectify their outcomes is akin to “forcing a square peg into a round hole” (p. 7). They suggest that instead of prioritising quantitative outputs and concrete changes in the research itself, impact should be thought of more in terms of experiential knowledge and changes in researchers’ attitudes [34–36]. In this framework, involvement is not primarily an intervention, but an everyday experience involving conversations between researchers and the public that support two-way learning. In their words, “Such conversations help to improve ideas, help researchers to make better choices and decisions, and help to solve their problems. This is part of the individual’s subjective experience of conducting research” [34: 7]. The approach they advocate fits well with what we heard in the interviews, and would serve to address researchers’ concerns about the ‘intangibility’ of impact and the impossibility of measuring it in the terms that funders require.

Clearly, our findings have various limitations that reflect the context in which the project was conducted. First, they reflect the experiences of a small sample of self-selected researchers linked to the NIHR Imperial BRC. All the researchers we interviewed had previously received support for public involvement through PERC, and it is likely that this context influenced who agreed to be interviewed and what they said. Boaz and colleagues [10] note, “NIHR funding for these flagship initiatives is accompanied by requirements to adhere to NIHR guidance on PPI” (p. 594). Although most health and medical researchers UK first encounter public involvement when applying for funding [14], NIHR has a more prescriptive approach to public involvement than other funders, which may have inflected the feedback we received.

Nevertheless, our project findings also point to the broader challenges of studying researchers’ experiences of public involvement, given the normative assumption of public involvement as a self-evident and unassailable good [23]. Evident was a degree of ‘moral accounting’ work [32] in participants’ responses to our questions about their motivations for public involvement.

Interviewees were clearly aware that their public involvement activities are expected to be driven more by a personal desire to learn from patients and the public than funders’ requirements for such, and this inevitably shaped their responses to some degree. This context means that without due care, those exploring the impact of public involvement on research are liable to receive rote narratives about public involvement that merely parrot what researchers are repeatedly told about its value.

We doubt it is a coincidence that the most senior academics we interviewed were the most open in their criticisms of public involvement, as they presumably would have had the least to lose in voicing their opinions freely. As Boylan and colleagues [12] note, researchers holding very critical views of public involvement often do not feel empowered to voice these views publicly. This illustrates that researchers’ experiences of public involvement is a morally charged topic – a point that needs to be more explicitly considered in future research and how data are analysed. If anything, this context leads us to suspect that our findings potentially understate researchers’ cynicism and ambivalence about the practice of public involvement.

## Conclusions

Our project suggests that funders’ requirements for public involvement have facilitated its implementation as normal practice in health and medical research, but at the expense of tokenism. In other words, tokenistic approaches to public involvement are an inevitable consequence of mandating it. This yields some benefits insofar as doing public involvement helps researchers understand its value, but it has also served to create a split between what researchers identify as ‘real’ and ‘organic’ public involvement, and its ‘artificial’ and ‘ratified’ versions. The consistent message in our interviews was the need for a nuanced, flexible approach to public involvement rather than the one-size-fits-all orientation favoured by funders.

Our project also suggests that tokenism cannot just be blamed on recalcitrant researchers unwilling to share power with patients and the public. Instead, if institutions and funders are serious about public involvement, then cultural shifts in these organisations are required so that public involvement doesn’t just become one more burden placed on already overburdened academics. It has been known for decades that public involvement requires time, resources and recognition. Thus, the most surprising aspect of our findings is how little things have changed on this front, despite the greater visibility of public involvement. As Boylan and colleagues [12] observe, this suggests that what is required is “a cultural change to challenge the tick-box approach that may have resulted from making PPI an operational requirement” (p. 729).

Finally, our project reaffirms the importance of new approaches to conceptualising impact that shift the focus from the research itself to the researchers who carry it out. Instead of seeing public involvement as a panacea for a variety of very different issues (e.g., research quality and impact, ensuring that the public’s voice is heard, guaranteeing that research is relevant to patients and the public), we believe that a more modest approach focusing on public involvement as a *dialogue* rather than an *intervention* may ultimately yield greater benefits. As the saying goes, all problems exist in the absence of a good conversation.

## Electronic Supplementary Material

Below is the link to the electronic supplementary material.

Supplementary Material 1

Supplementary Material 2

## Data Availability

To protect participants’ privacy, data from individual participants are not publicly available.
