# Supplementary material for: “If the funders said to me that I have to dress in orange, I would dress in orange”: findings from qualitative interviews on researchers’ experiences of public involvement
Source: Res Involv Engagem. 2026 Jul 3;12:106. doi: 10.1186/s40900-026-00901-9 (PMC13330384; doi:10.1186/s40900-026-00901-9)
Supplement: Supplementary file 1 — Supplementary Material 1 [file 40900_2026_901_MOESM1_ESM.docx]

**Appendix 1 – interview guide**

**Could you briefly tell me about the research that you do?**

**What do you understand by the term ‘public involvement’?**

**How have you integrated public involvement into your research?**(note what they say AND don’t say)

- Who was involved and how did you get in touch with them?
- How were they involved?
- When were they involved – at specific stages or continuously?
- What were you hoping to achieve by involving them in your research?
- Intended plans vs. actual outcomes?

**Has public involvement changed your research project at all? If so, in what ways?**

- Are there any specific examples you can think of?
- Were the impacts communicated to the public involvement participants/communities involved?

**How important did you find public involvement in your research?**

**Did you receive any formal training on how to do public involvement?**

- Did you receive any (other) support regarding public involvement in your research?

**What was your experience with public involvement like?**

- In your experience, what are the positives and challenges of public involvement?
- Have your views on public involvement changed over time (over course of research or career)?
- If anything, what would you do differently if you did a public involvement project again? / Are there any changes you would make to the public involvement process?

**Is there anything else you’d like to add or mention?**
