# Supplementary material for: “If the funders said to me that I have to dress in orange, I would dress in orange”: findings from qualitative interviews on researchers’ experiences of public involvement
Source: Res Involv Engagem. 2026 Jul 3;12:106. doi: 10.1186/s40900-026-00901-9 (PMC13330384; doi:10.1186/s40900-026-00901-9)
Supplement: Supplementary file 2 — Supplementary Material 2 [file 40900_2026_901_ReviewerReport_V0_R1.pdf › 40900_2026_901_ReviewerReport_V0_R1.pdf]

- **Anne Wettergren Karlsson | Reviewer recommendation for Editor : Revise**

20-Feb-2026 14:56:06

FEEDBACK FOR AUTHOR(S)

Thank you for an interesting glimpse into the status of PPI in a research center in the UK. Please see further comments in the reviewer comments file.

FEEDBACK FOR EDITOR

Your recommendation Revise Is the study design appropriate to answer the research question (including the use of appropriate controls), and are the conclusions supported by the evidence presented? No, but these points can be addressed with revisions Comments: I think the article needs to be rewritten as a discussion piece or a case study. I dont think the analysis or methods are strong enough as a research piece. I have provided further comments in the reviewer file. Please rate the novelty of the research on a scale of 1-5 (1=not at all novel and 5=extremely novel)? 3 Comments: As it is written now, it is mostly for a UK audience - but it has relevance for an international audience, if providing some more context. Is the presentation of the work clear, with regards to language and grammar? Acceptable Does the title of this paper clearly reflect its content? No Comments: I think the quote does not reflect the main results of the article. I have made an alternative suggestion in the reviewer file. Does the abstract sufficiently reflect the content? Yes Are the keywords representative of the research? Yes Does the introduction present the purpose of the investigation and is the purpose supported by the pertinent literature? Yes Are the methods described sufficiently to allow the study to be repeated by other parties? No Comments: There are a description of what was done, but no theory used or explanation why it was not used. Are Research Resource Identifiers (RRID) included where applicable? Not applicable Is the use of statistics and treatment of uncertainties appropriate? Not relevant to this manuscript Are the images in this manuscript (including electrophoretic gels and blots) free from apparent manipulation? Not applicable Are the table and figures well designed and necessary? Yes Are the references provided appropriate and up to date? Yes

Confidential Comments to the Editor No further comments

- **Edilene Lopes | Reviewer recommendation for Editor : Reject**

16-Feb-2026 04:49:17

FEEDBACK FOR AUTHOR(S)

I have not reviewed the manuscript as I have ethical concerns about its suitability for publication. I have mentioned those in my comments to the editors.

FEEDBACK FOR EDITOR

Your recommendation Reject Is the study design appropriate to answer the research question (including the use of appropriate controls), and are the conclusions supported by the evidence presented? No, there are fundamental issues that cannot be addressed Comments: I sent the email below to the journal editors and was waiting for a response before proceeding with the review. So, I will copy it here and wait for further instructions. Dear Richard Stephens and Sophie Staniszewska, I received the email below requesting my review of a manuscript for the Research Involvement and Engagement journal. When I started reading the manuscript, I could see that, despite involving human participants, the project has not undergone ethical review, as it was initially classified as a quality and assurance project within the institution where it was conducted. The authors stated that they sought permission from interviewees to publish the paper; however,

this permission was retrospective, and the project does not appear to meet the ethical standards expected of research that has undergone an ethical review. This situation seems to be inconsistent with the BMC editorial policies on “ethics and consent,” which specify: “Ethics approval Research involving human participants, human material, or human data, must have been performed in accordance with the Declaration of Helsinki and must have been approved by an appropriate ethics committee. A statement detailing this, including the name of the ethics committee and the reference number where appropriate, must appear in all manuscripts reporting such research. If a study has been granted an exemption from requiring ethics approval, this should also be detailed in the manuscript (including the name of the ethics committee that granted the exemption). Further information and documentation to support this should be made available to the Editor on request. Manuscripts may be rejected if the Editor considers that the research has not been carried out within an appropriate ethical framework. In rare cases, the Editor may contact the ethics committee for further information. Retrospective ethics approval If a study has not been granted ethics committee approval prior to commencing, retrospective ethics approval usually cannot be obtained, and it may not be possible to consider the manuscript for peer review. The decision on whether to proceed in such cases is at the Editor's discretion.” (Editorial policies | BMC Journals). Given this context, I would like to ask about how best to proceed. Should I continue with the review process for this manuscript, or would you prefer to contact the authors directly regarding these ethical concerns? Kind regards, Edilene Edilene Lopes McInnes. PhD, MPhil (Public Health) Research Fellow | School of Humanities, College of Creative Arts, Design and Humanities ADELAIDE UNIVERSITY Kaurna Yerta Level 7, Napier Building City East Campus | SA 5005 T: +61 8 8313 5296 E: edilene.lopesmcinnes@adelaide.edu.au https://researchers.adelaide.edu.au/profile/edilene.lopesmcinnes Latest publication: Commoning contingent resources: constructing an Australian stem cell registry Adelaide University respectfully acknowledges the Kaurna, Boandik and Barngarla First Nations Peoples and their Elders past and present, who are the First Nations’ Traditional Owners of the lands that are home to our campuses located in Adelaide, Magill, Mawson Lakes, Waite, Roseworthy, Mount Gambier and Whyalla. image Australian University Provider Number PRV14404 CRICOS Provider Number 04249J -- IMPORTANT: This email may be privileged and/or confidential, and the sender does not waive any related rights and obligations. Any distribution, use or copying of this email or the information it contains by other than an intended recipient is unauthorised. If you receive this email in error, please advise me (by return email or otherwise) immediately. --- image Please rate the novelty of the research on a scale of 1-5 (1=not at all novel and 5=extremely novel)? Not relevant for this manuscript Is the presentation of the work clear, with regards to language and grammar? Not suitable for publication unless extensively edited Does the title of this paper clearly reflect its content? Yes Does the abstract sufficiently reflect the content? Yes Are the keywords representative of the research? Yes Does the introduction present the purpose of the investigation and is the purpose supported by the pertinent literature? Yes Are the methods described sufficiently to allow the study to be repeated by other parties? No Are Research Resource Identifiers (RRID) included where applicable? Not applicable Is the use of statistics and treatment of uncertainties appropriate? Not relevant to this manuscript Are the images in this manuscript (including electrophoretic gels and blots) free from apparent manipulation? I am not able to assess this Are the table and figures well designed and necessary? Yes Are the references provided appropriate and up to date? Yes

Confidential Comments to the Editor Dear Richard Stephens and Sophie Staniszewska, I received the email below requesting my review of a manuscript for the Research Involvement and Engagement journal. When I started reading the manuscript, I could see that, despite involving human participants, the project has not undergone ethical review, as it was initially classified as a quality and assurance project within the institution where it was conducted. The authors stated that they sought permission from interviewees to publish the paper; however, this permission was retrospective, and the project does not appear to meet the ethical standards expected of research that has undergone an ethical review. This situation seems to be

inconsistent with the BMC editorial policies on “ethics and consent,” which specify: “Ethics approval Research involving human participants, human material, or human data, must have been performed in accordance with the Declaration of Helsinki and must have been approved by an appropriate ethics committee. A statement detailing this, including the name of the ethics committee and the reference number where appropriate, must appear in all manuscripts reporting such research. If a study has been granted an exemption from requiring ethics approval, this should also be detailed in the manuscript (including the name of the ethics committee that granted the exemption). Further information and documentation to support this should be made available to the Editor on request. Manuscripts may be rejected if the Editor considers that the research has not been carried out within an appropriate ethical framework. In rare cases, the Editor may contact the ethics committee for further information. Retrospective ethics approval If a study has not been granted ethics committee approval prior to commencing, retrospective ethics approval usually cannot be obtained, and it may not be possible to consider the manuscript for peer review. The decision on whether to proceed in such cases is at the Editor's discretion.” (Editorial policies | BMC Journals). Given this context, I would like to ask about how best to proceed. Should I continue with the review process for this manuscript, or would you prefer to contact the authors directly regarding these ethical concerns? Kind regards, Edilene Edilene Lopes McInnes. PhD, MPhil (Public Health) Research Fellow | School of Humanities, College of Creative Arts, Design and Humanities ADELAIDE UNIVERSITY Kaurna Yerta Level 7, Napier Building City East Campus | SA 5005 T: +61 8 8313 5296 E: edilene.lopesmcinnes@adelaide.edu.au https://researchers.adelaide.edu.au/profile/edilene.lopesmcinnes Latest publication: Commoning contingent resources: constructing an Australian stem cell registry Adelaide University respectfully acknowledges the Kaurna, Boandik and Barngarla First Nations Peoples and their Elders past and present, who are the First Nations’ Traditional Owners of the lands that are home to our campuses located in Adelaide, Magill, Mawson Lakes, Waite, Roseworthy, Mount Gambier and Whyalla. image Australian University Provider Number PRV14404 CRICOS Provider Number 04249J -- IMPORTANT: This email may be privileged and/or confidential, and the sender does not waive any related rights and obligations. Any distribution, use or copying of this email or the information it contains by other than an intended recipient is unauthorised. If you receive this email in error, please advise me (by return email or otherwise) immediately. -- image

- **Nicola Lloyd | Reviewer recommendation for Editor : Revise**

06-Feb-2026 16:09:23

FEEDBACK FOR AUTHOR(S)

Thank you for inviting me to review this manuscript on NIHR researchers’ experiences of public involvement and the reasons they do it. This is a well-written, well-structured article on an important topic that will be of interest to multiple stakeholders. The discussions and conclusions are very strong and relevant to current debates surrounding public involvement in research and health. The authors have appropriately acknowledged some limitations with the study design and recruitment, and the findings remain worthwhile and relevant despite these. Some suggestions for improvement are outlined in the attached document.

FEEDBACK FOR EDITOR

Your recommendation Revise Is the study design appropriate to answer the research question (including the use of appropriate controls), and are the conclusions supported by the evidence presented? Yes Please rate the novelty of the research on a scale of 1-5 (1=not at all novel and 5=extremely novel)? 4 Comments: This paper explores nuances and adds new knowledge to ongoing debates about the rationales for public involvement and the influences of policy and funder requirements. Is the presentation of the work clear, with regards to language and grammar? Acceptable Does the title of this paper clearly reflect its content? Yes Does the

abstract sufficiently reflect the content? Yes Are the keywords representative of the research? Yes Does the introduction present the purpose of the investigation and is the purpose supported by the pertinent literature? Yes Are the methods described sufficiently to allow the study to be repeated by other parties? No Comments: I have made some comments in the report that could improve the transparency of the data analysis. Are Research Resource Identifiers (RRID) included where applicable? Not applicable Is the use of statistics and treatment of uncertainties appropriate? Not relevant to this manuscript Are the images in this manuscript (including electrophoretic gels and blots) free from apparent manipulation? Not applicable Are the table and figures well designed and necessary? Yes Are the references provided appropriate and up to date? Yes

Confidential Comments to the Editor Thank you for the opportunity to review this manuscript, which is well written and should be of interest to a variety of stakeholders and readers of the journal. I recommend it is accepted and have made minor suggestions for improving it in my report. I would also like to note that I am relatively new to reviewing and would welcome any feedback you might have on this review. If you have any suggestions to improve my reviewing skills, they would be gratefully appreciated.

- **Bronwen Merner | Reviewer recommendation for Editor : Reject**

02-Feb-2026 13:27:04

FEEDBACK FOR AUTHOR(S)

This study does not have ethics approval. While the authors have sought to explain why the study changed from a quality assurance to a research project, some interviewees have been very frank in their comments. I am unsure that the same frankness would have been achieved if the interviewees knew, from the outset, that the results would be published. I am also concerned about the possibility of re-identification of the participants given they were drawn from a small pool. The authors make some interesting points in the Discussion and I think overall, they have done an adequate job. But I feel the ethical implications of not having the study reviewed by an ethics committee and receiving post-interview consent mean the study is not acceptable for publication in this journal.

FEEDBACK FOR EDITOR

Your recommendation Reject Is the study design appropriate to answer the research question (including the use of appropriate controls), and are the conclusions supported by the evidence presented? No, there are fundamental issues that cannot be addressed Comments: See comments to author. Please rate the novelty of the research on a scale of 1-5 (1=not at all novel and 5=extremely novel)? Not relevant for this manuscript Is the presentation of the work clear, with regards to language and grammar? Acceptable Does the title of this paper clearly reflect its content? Yes Does the abstract sufficiently reflect the content? Yes Are the keywords representative of the research? Yes Does the introduction present the purpose of the investigation and is the purpose supported by the pertinent literature? Yes Are the methods described sufficiently to allow the study to be repeated by other parties? Yes Are Research Resource Identifiers (RRID) included where applicable? Yes Is the use of statistics and treatment of uncertainties appropriate? Not relevant to this manuscript Are the images in this manuscript (including electrophoretic gels and blots) free from apparent manipulation? Not applicable Are the table and figures well designed and necessary? Yes Are the references provided appropriate and up to date? Yes

Confidential Comments to the Editor
