# Supplementary material for: “If the funders said to me that I have to dress in orange, I would dress in orange”: findings from qualitative interviews on researchers’ experiences of public involvement
Source: Res Involv Engagem. 2026 Jul 3;12:106. doi: 10.1186/s40900-026-00901-9 (PMC13330384; doi:10.1186/s40900-026-00901-9)
Supplement: Supplementary file 2 — Supplementary Material 2 [file 40900_2026_901_ReviewerReport_V1_R1.pdf › 40900_2026_901_ReviewerReport_V1_R1.pdf]

Title: “If the funders said to me that I have to dress in orange, I would dress in orange”: Findings from qualitative interviews on researchers’ experiences of public involvement

Version: 1 **Date:** 15 Apr 2026

Reviewer: Anne Wettergren Karlsson

Reviewer's report:

I was also part of the initial review committee, and I was quite surprised to see comments regarding ethical issues of this study. I think you have handled them well, and added descriptions which have justified your approach and work.

I am satisfied with the changes to the script you have made on the basis of my previous comments, and I read the script now as much stronger, coherent and transparent for an international audience. So, I appreciate the changes. - I still do not agree with the title - but it is just my opinion, you are certainly entitled to your opinion too :-)

I have no further requests at this point. Good luck with the publication.
